# Thermal Analysis of Infrared Irradiation-Assisted Nanosecond-Pulsed Tumor Ablation

**DOI:** 10.1038/s41598-020-62017-8

**Published:** 2020-03-20

**Authors:** James Hornef, Chelsea M. Edelblute, Karl H. Schoenbach, Richard Heller, Siqi Guo, Chunqi Jiang

**Affiliations:** 1 Frank Reidy Research Center for Bioelectrics, Old Dominion University, Norfolk, VA 23508 USA; 2 Department of Electrical & Computer Engineering, Batten College of Engineering & Technology, Old Dominion University, Norfolk, VA 23529 USA

**Keywords:** Lung cancer, Biomedical engineering, Electrical and electronic engineering, Fibre lasers

## Abstract

Nanosecond Pulsed Electric Fields (nsPEF) have the potential to treat a variety of cancer types including melanoma, pancreatic and lung squamous cancers. Recent studies show that nsPEF-based cancer therapy may be improved further with the assistance of moderate heating of the target. A feedback-looped heating system, utilizing a 980-nm fiber optic laser, was integrated into nsPEF electrodes for tumor ablation. The laser beam profile was determined to be Gaussian using a knife-edge technique. Thermal properties of the biological target were evaluated based on the treatment area, penetration depth and thermal distribution due to laser irradiation with or without nsPEF. Synergistic effects between nsPEF and the moderately elevated temperature at the target was observed, resulting in enhanced overall survival tumor regression up to 50% in the treatment of lung squamous cell cancer in mice.

## Introduction

The use of Nanosecond Pulsed Electric Fields (nsPEF) in biological studies is known for providing a non-thermal treatment alternative due to its unique biological effects such as apoptosis in cells and tumors^1–3^, activation of signaling pathways used in cell death^4,5^ and transferring specific genes into cells through cell membrane permeation^6^. NsPEF has also been shown to be a novel way to treat several cancer types including breast cancer^7^, hepatocellular carcinoma^8^, and squamous cell carcinoma^2^, through Irreversible Electroporation (IRE). A key aspect to the effectiveness of this nsPEF treatment is how the fields are delivered. The method of delivery not only affects the electrical parameters, such as the electric field strength, but also the electric field distribution, which determines the effective area that is being treated.

Two of the more common apparatuses used in nsPEF treatment of cancer are the parallel-plate electrodes and the needle-array electrodes. In the case of parallel-plate electrodes, the separation distance between the two plate electrodes must be smaller than the minimal transverse dimension of the plates so that the electric field applied is uniformly distributed over the treating target that is placed between the plates. The parallel-plate electrodes have been used for treating cutaneous tumors^9^, and are also possible to be used for open surgery where the treating area is easily accessible. However, since the electric field strength depends on the separation distance between the plates, the gap distance is typically fixed by design, allowing only a small range of tumor sizes to be effectively treated. Air gaps between the treating tissue and plates due to tumor sizes being too smaller or incomplete treatment due to larger tumors make parallel-plate electrodes ill-suited for variable treatment area sizes or deep tissue tumors^10^.

In the case of needle-array electrodes, an electric field is generated using the needles as the anodes and cathodes, causing the distribution of the electric field to be dependent on the organization of the needles. This electrode type allows for the treatment of deep-seated tumors as well as tumors larger than the needle spacing, through tissue insertion and multiple treatment sites respectively^11^. Unfortunately, a significant limitation to this electrode type is the non-uniformity of the electric field. When applying the electric field using a needle-array electrode, the field strength is strongest near the needles, and decreases substantially as the field moves away, resulting in a highly non-uniform electric field distribution and uneven treatment results. Such non-uniformity in field distribution requires a higher intensity electric field to be used in order to achieve ablation results for larger sites, which poses problems including thermal tissue damage caused by electric breakdown or overstimulation in deep tissue at the areas where the fields are the strongest^8^. An ideal delivery apparatus would have the versatility of the needle array electrode with the uniformity of the parallel plate electrode. So, a new delivery apparatus needs to be created to achieve this.

A promising solution for the non-uniform electric field distribution provided by needle-array electrodes is to add moderate heating (e.g. ~43 °C for 2 min) during treatment. Previously, synergistic effects of local temperature enhancements on cellular responses to intense nsPEF, in additional to the electrical effect, were suggested using a molecular dynamics modeling^12^. This is important since these localized thermal contributions to bio-processes could be beneficial but the globally averaged temperature effects might still seem negligible^12^ (and hence the nsPEF treatment can still be categorized as non-thermal). Cells or tumor exposure to 43 °C has been demonstrated to enhance the toxicity of chemotherapeutic drugs^13^ and radiation^14^. The combination of moderate heating and PEF for treatment could prove to be beneficial. In fact, synergistic effects between moderate heating and PEF have already been demonstrated in several different applications, including enhancing the treatment of *Lactobacillus brevis* cell cultures *in vitro*^15^, enhancing the effectiveness of gene electrotransfer *in vivo*^16^ and increasing the efficacy of pancreatic cancer treatment using microsecond PEF (μsPEF), both *in vitro* and *in vivo*^17^. In all these cases, different heating sources were used, such as heat blocks, contact heaters and fiber optic lasers. There are several heating options available for this integration, such as joule heating^18^, radio frequency heating^19^ and laser heating^20,21^. For our experiments, we chose to implement laser heating, using the same system as the pancreatic cancer treatment^17^, due to its ease of integration as well as allowing for fine control over the heating location, distribution and intensity.

In this paper, a 4-needle electrode system that integrates an infrared temperature sensor, a fiber optic laser and an automated heating control system was developed. This electrode system can monitor and maintain a user specified temperature while delivering high voltage nsPEF in real time. Using this system, the effects of laser heating on the thermal or optical properties of tissue, such as absorption and transmission, were analyzed. The synergistic effects of moderate heating and nsPEF on the treatment of lung squamous cell tumors *in vivo* were evaluated.

## Results and Discussion

### Thermal properties of pig skin and the heat distribution

**Figure 1:**
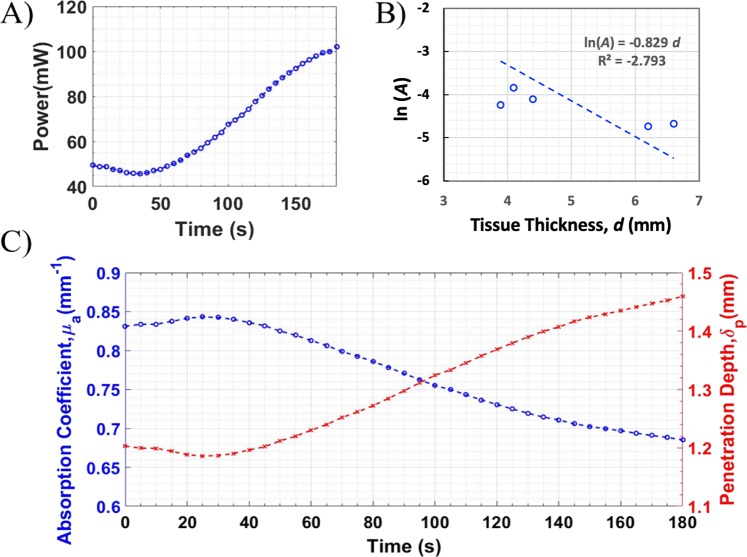
Time-dependent absorption coefficient of pig skin under constant laser irradiation at 2.3 W. (**A**) A transmitted light intensity over time for a tissue sample of 3.9 mm thickness. (**B**) An example transmittance in natural log versus tissue thickness at 30 seconds of heating and a zero-intercept linear fit. The slope of this fit equals the equivalent absorption coefficient at that time. (**C**) Equivalent absorption coefficient and laser penetration depth versus time. Data shows that the optical properties of the tissue samples begin to deteriorate after 30 seconds of laser exposure, allowing more laser light to penetrate the sample.

Figure 1 shows the equivalent absorption coefficient and penetration depth of the pig skin, based on the tested tissue samples, as functions of time obtained by following the Beer-Lambert’s Law, described in the later section under Materials and Methods and Eq. (4). An example transmitted light as a function of time for a tissue sample with a thickness of 3.9 mm is shown in Fig. 1A. To obtain the absorption coefficient at a given treatment time, five samples with various thickness ranging from 3.9 to 6.8 mm were used. Figure 1B shows the transmittance ***A*** in natural log with respect to the sample thickness *d*. A linear fit with zero-intercept was applied to this relationship (Fig. 1B), the slope of which equals to the equivalent absorption coefficient of the pig skin at the given time. The zero-intercept linear fit assumes that when the skin thickness reduces to zero, light is completely transmitted to the detector or the transmittance equals to 1 (*i.e*. ln(***A***) = 0). Note that the deviation of the fitting is relatively large, which is possibly due to that the hypodermal layer has significantly different absorption and scattering characteristics from those of the epidermal and dermal layers^22^. The 3-mm sections of pig skin had a minimal hypodermal layer, leaving primarily the dermal and epidermal layers. Any thicker skin sample would contain a larger hypodermal layer of tissue. Nevertheless, the equivalent absorption coefficient, ${\mu }_{a}$, is an approximate coefficient of the sample group, and can be used to represent the thermal property changes of the pig skin due to laser irradiation. Since the penetration depth ${\delta }_{p}$ is the inverse of ${\mu }_{a}$ (see the details in the later section under Materials and Methods and Eq. (5)), the thermal properties, ${\mu }_{a}$ and ${\delta }_{p}$, of the pig skin as functions of time were obtained, as shown in Fig. 1C.

There were significant changes observed in the equivalent absorption coefficient of the pig skin after prolonged laser exposure. Within the first 30 seconds, the change in the absorption coefficient, ${\mu }_{a}$, was small and slightly increased over time. After 30 seconds of laser heating, ${\mu }_{a}$ started decreasing and was 82% of its maximal value after 3 min-heating. Prolonged exposure to heat is known to denature tissue, which would alter its optical properties over time^23^. Additionally, the thicker tissue samples (e.g. above 4.1–6.5 mm) had a notable drop in the initial absorption coefficient. In this data set, the thickness of epidermal and dermal layers of skin remained constant, with the thinner samples, e.g. 3.9-mm samples, consisting of only these two layers. Samples thicker than this contained a fatty layer of tissue, which is known to have a much higher scattering coefficient than the other two layers^12,22,24^. Analysis of the effect of the fatty layer of tissue on the total absorption coefficient revealed an inverse relationship between the two, where fatty layers ranging from 0.2–2.5 mm caused a 6–22% drop in total absorption coefficient respectively. These results are comparable with the *in vivo* and *in vitro* optical studies performed on human skin^25,26^. Moreover, while thicker fatty layers do not alter this relationship, it does shift it to a lower starting value. Results show that samples with larger fatty layers will require a longer exposure to the laser for complete heating.

**Figure 2:**
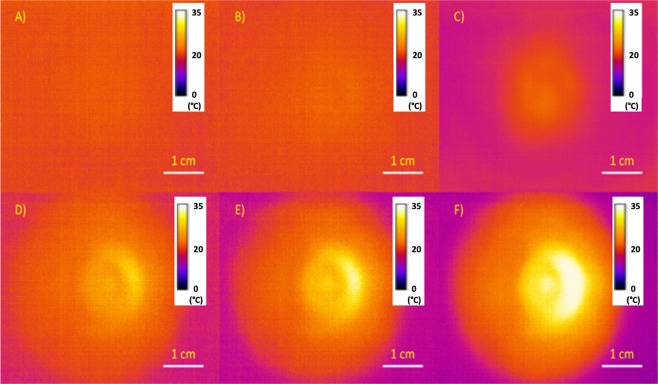
Time-dependent thermal images of a 4.1 mm sample of pig skin under constant laser irradiation at 2.3 W. Images are shown at laser exposure time = 0 (**A**), 30 seconds (**B**), 60 seconds (**C**), 90 seconds (**D**), 120 seconds (**E**), and 180 seconds (**F**). The color scale shows the surface temperature in °C. The ring-shaped heat distribution that begins to appear around the edge of the heat distribution is due to the use of the camera lens. Within the first 30 seconds after laser heating, there was no noticeable change in the heat distribution. The same spatiotemporal development was observed for all samples, regardless of thickness.

The temperature distribution observed at the rear of the sample followed the absorption coefficient results described above. For the first 20–30 seconds of exposure, there was no heat increase observed. However, after the ~30-second delay, a steady increase in temperature and heat distribution was observed over time (Fig. 2). This can be attributed to the decline in absorption coefficient after 30 seconds of laser exposure. The longer the sample was exposed to the laser light, the more the absorption coefficient decreased, allowing more laser light to transmit through the sample. This trend was observed for all thickness samples, the only difference being that the thicker samples took slightly longer to reach the peak temperature of 35 °C at the back of the sample.

In addition, after the tissue sample became mostly transparent to the laser irradiation, e.g. after 120 seconds heating, the distribution of the temperature, recorded by the thermal camera, revealed a Gaussian profile, matching the profile of the laser beam determined by the knife edge technique. The area of the thermal distribution at the peak laser irradiation based on the beam profiling measurements was found to be about 2 cm^2^, which is less than the area between the needles of the electrode, 3.5 cm^2^. However, this area only accounts for the peak temperature reading of 38 °C. The entire heating area was determined to be ~4 cm^2^, which is enough to cover the entire region between the needles. This suggests that the spot size of the laser fiber is sufficient for distributive and penetrative heating. It is important to note that the thermal distribution was only measured through the rear of the sample. The area of heating on the surface of the tissue directly exposed to the incident laser beam is expected to be larger than that away from the directly exposed surface in an ideal thermal dynamics model^27^.

### Effectiveness of combinational treatment on KLN205 tumors in mice

**Figure 3:**
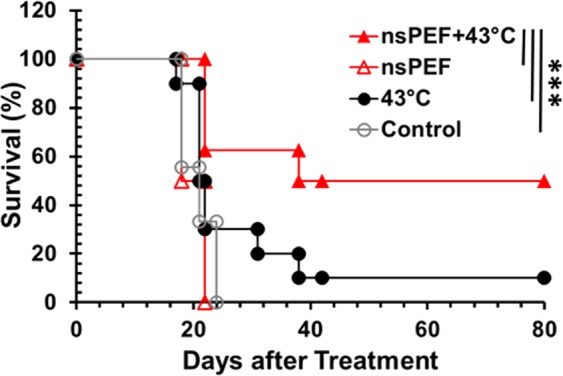
Kaplan Survival Curves of *in vivo* treatment. Mice were treated on day 0 with nsPEF alone, laser heating alone or nsPEF + laser heating. Control: no treatment (n = 9); 43 °C: laser heating only (n = 10); nsPEF: nsPEF only (n = 8), nsPEF parameters: 100 ns pulse width, 5 pulses per second, 600 pulses and electric field strength of 14 kV/cm; nsPEF + 43 °C: tumor preheated using laser heating to 43 °C and maintained for 1 minute before PEF was applied (n = 8). ***p <0.001 for nsPEF + 43 °C vs Control, laser only (43 °C) or nsPEF (Log-rank test).

Prior to the combination treatment, a pulse intensity-escalating study was performed and demonstrated nsPEF with 14 kV/cm was below the threshold of tumor ablation. There was no (0/5) complete tumor ablation resulting from 14 kV/cm nsPEF, however, 22 kV/cm and 30 kV/cm nsPEF achieved 62.5% (5/8) and 80% (4/5) complete tumor regression, respectively. Other pulse parameters were 100 ns pulse duration, 5 Hz and 600 pulses, for all three intensities of nsPEF. In order to demonstrate that preheating tumor can reduce the threshold of nsPEF, we adopted 14 kV/cm nsPEF for the combination treatment. During treatment, an average surface temperature of 43–44 °C was achieved, translating to a 42 °C internal temperature after the pre-heating was complete. Rise time of the heating depends heavily on the condition of the tumor, such as its shape and if there are ulcerations or cellular necrosis present. The Kaplan-Meier survival curves (Fig. 3) show that 14 kV/cm nsPEF treatments alone had no effect on the tumor growth, with a median survival of 18 days. Animals with tumors treated with only 43 °C heating did slightly better but not statistically significant, with a median survival of 21 days and just one mouse remaining until day 80. However, when combining these two treatments, survival was significantly extended to 38 days. Importantly, 50% of mice survived up to 80 days after treatment, showing a synergistic effect between the moderate heating and the nsPEF. Moreover, preheating tumor appeared to improve the efficacy of 22 kV/cm nsPEF as well. The combination of heat and 22 kV/cm nsPEF achieved 75% (6/8) complete tumor regression in contrast to 62.5% (5/8) for nsPEF alone. The treatment efficacies of different nsPEF conditions with or without moderate heating were compared in Table 1. Although moderate heating enhanced tumor shrinking under 22 kV/cm nsPEF treatment, it did not show a significant survival improvement. This implies that moderate heating had more impact on animal survival when it was combined with suboptimal nsPEF conditions, or it seemed more valuable at circumstances when a very-high electric field could not be administered.

**Table 1:** Comparison of treatment effectiveness by nsPEFs with or without moderate heating (MH). When heating was applied, the surface temperature was maintained at 43 °C.

nsPEF Treatment Conditions: (100 ns, 5 Hz, 600 pulses)	Tumor Size at Day 11 (Mean ± SE)	Statistics (T-test)	Complete Regression	Statistics (LogRank)
14 kV/cm	195.1 ± 7.1 (mm^3^)	p = 0.137	0% (0/8)	p < 0.001
14 kV/cm with MH	129. ± 12.6 (mm^3^)	p = 0.137	50% (4/8)	p < 0.001
22 kV/cm	121.2 ± 10.0 (mm^3^)	p = 0.056	62.5% (5/8)	p > 0.05
22 kV/cm with MH	47.9 ± 7.4 (mm^3^)	p = 0.056	75% (6/8)	p > 0.05

The above findings demonstrate that moderate heating (43 °C for ≤2 min) or nsPEF alone (14 kV/cm) has a minimal or no effect on tumor ablation but the combination therapy achieves a significant tumor ablation. Similar results have been shown using the 4-needle electrode orientation and laser heating with microsecond pulses using Pan02 pancreatic cancer cells, suggesting that the addition of heat to PEF treatments have beneficial effects^17,28^. Moreover, our previous numerical analyses of electric field distribution in similar electrode configurations support that preheating tumor was able to greatly reduce the threshold of electric field strength for tumor ablation^29^. The use of a range of electric parameters and the requirement for different electric field strengths for *in vitro* 3D and *in vivo* pancreatic tumor models to achieve comparable results were reported previously^17^. These data support our hypothesis that the addition of moderate heating to this nsPEF treatment produces synergistic effects. However, the diverse physical properties and shape deformities of the tumors treated within the *in vivo* procedures resulted in less than ideal conditions for treatment. For example, the presence of ulcerations and central necrosis on the surface of the tumor caused uneven treatment effects, resulting in recurrence of the tumors post treatment. This could be due to these physical deformities causing drastic changes in the optical properties of the tumor, including penetration of the laser heating as well as absorption of the heat into the tumor. Both the absorption coefficient measurement and temporal development of the thermal image of pig skin showed the penetration depth increases after 60 s heating and reached up to 1.25 times of the initial value after 180 s heating. In this *in vivo* mouse model, a tumor thickness of 4–5 mm below the surface was able to achieve 42 °C after 60-s laser irradiation, and hence the heating condition was expected to reach 5–6 mm by the end of the 2-min nsPEF + 43 °C treatment. As the surface temperature was used as the sole reference for laser heating modulation, smaller tumors may have had heating over a greater volume in normal tissue compared to bigger tumors. For treatment of bigger tumors, the pretreatment heating time would need to be increased in order to achieve the same temperature underneath the surface. Although no negative effect of temperature elevation up to 43 °C were detected in this study, other effects such as immune cell stimulation could contribute to the combined effects beneficially. Further testing is required to optimize the treatment standards for these imperfect targets, as well as improve the optical property monitoring before and after treatment before making completely rigorous conclusions.

In summary, a detailed thermal analysis of pig skin under infrared laser irradiation was presented. This analysis provides important and necessary information for the next-step clinical studies and is critical for the technology development and assessment for future clinical trials. Both absorption coefficient and penetration depth are functions of heat exposure time. The time-dependent thermal properties of the pig skin suggest the necessity of a feed-back controlled heating scheme as well as the limitation of the heating (with the measurement of the penetration depth). As a feasibility demonstration, an integrated nsPEF needle-array electrodes with infrared heating system was applied to KLN205 tumors in mice, and the synergistic effects between moderate heating of the target and nsPEF were observed. These results suggest that the use of controlled, moderate heating increases the effectiveness of nsPEF treatment by increasing the effective treatment area and potentially reducing the required electric field strength needed for complete tumor elimination. This is important since reduction of the electric field intensity can lead to a much safer treatment for the subject. Based on the beam profiling and thermal analysis study, the inherent non-uniformity in the laser irradiation power and the variations in the thermal property of tissue, a spatial distribution of the temperature over the target tissue are expected and the temperature of 43 °C was only an average surface temperature. After all, further optimization and testing are required to increase the heat distribution and penetration, as well as maintaining accurate temperature sensing and control. In addition, the thermal analysis was performed for pig skin, which is closer to human skin than *in vitro* and *in vivo* mouse models. Hence, the findings of the thermal properties are more applicable to future clinical uses. The demonstrative *in vivo* study is additional supportive evidence for the feasibility of this technology.

## Materials and Methods

### Controlled infrared heating system overview

**Figure 4:**
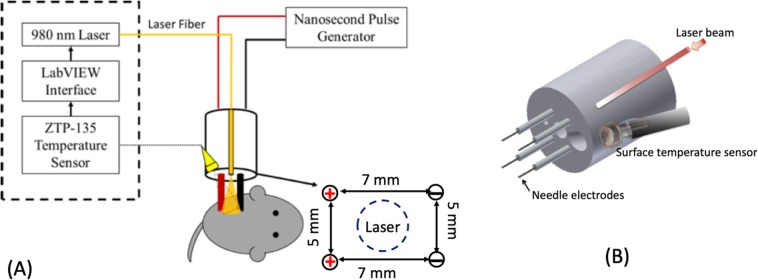
System Overview. Treatment set-up (**A**) for the *in vivo* study and the needle electrode array where the surface temperature sensor and laser fiber optic were integrated into the Teflon housing (**B**). The thermopile collects temperature readings in real time and sends them to a LabVIEW interface, which checks the current temperature reading against the ideal and adjusts the laser’s status accordingly. The LabVIEW interface also shows the temperature trend over the entire experiment and allows the user to manually disable the laser at any time. The dimensions of the needle electrode and the target area of the laser are also shown in (**A**).

The needle electrode array was constructed using a solid Teflon cylinder (2 cm in diameter and 2.5 cm long). Four 25-gauge needles of 1.5 cm length were used as the leads for the electric field and were organized in a 5 ×7 mm rectangle array in the center of the Teflon cylinder, as shown in Fig. 4. The electrode polarity was arranged such that the electrodes with the same polarity are 5 mm apart and the opposite 7 mm apart. The electrodes of the same polarity were electrically connected through the back of the Teflon cylinder, so that the voltage potential between the opposite electrodes was applied simultaneously. The laser fiber was fixed in the center of the electrode, radiating the central area of the target where the electric field strength is relatively weak. Ethylene tetrafluoroethylene (ETFE) tubing was used to cover 1.2 cm of the needles, leaving 3 mm exposed, to reduce the occurrence of electrical breakdown and helped to shield the integrated electronics from the generated electrical noise.

**Figure 5:**
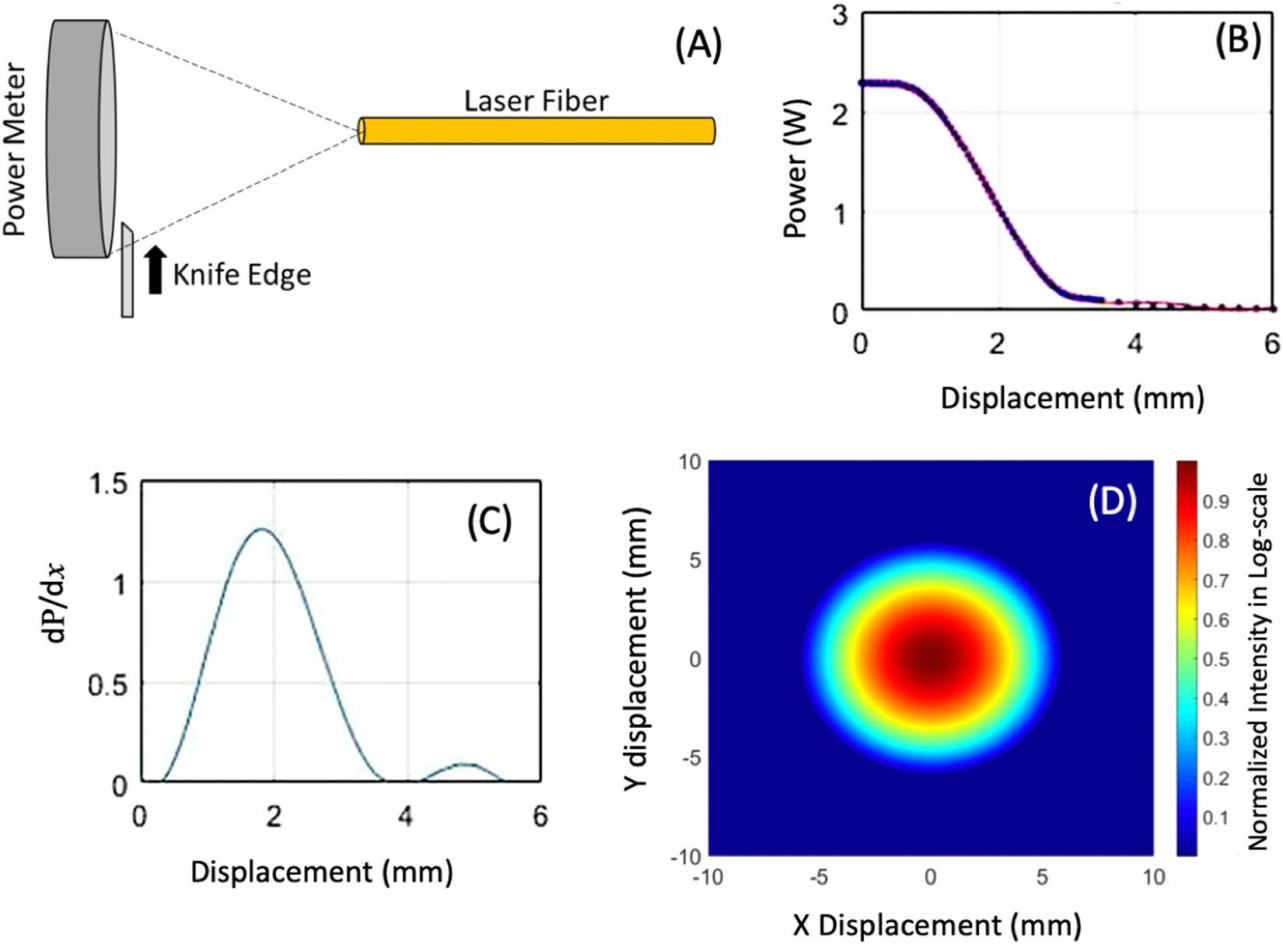
Determination of the laser beam profile using a knife-edge technique. (**A**) Schematic of a knife-edge technique; (**B**) Measured irradiation power as a function of the displacement of the knife-edge for a gap distance of 2 cm between the laser fiber and the power meter, along with a polynomial fit; (**C**) Derivative on the irradiation power from (**B**) over the displacement *x*, fitted to a 2^nd^ order Gaussian profile; The 1/*e*^2^ radius is 2.7-mm and was determined by taking the width of this profile at 13.5% of the peak value. (**D**) A 2D representation of the beam profile acquired in (**B**), shown in a log-scale of the normalized intensity. Note that the emission intensity relates to the incident energy of the laser beam, and the surface temperature of the target may vary depending on the thermal properties of the target.

The controlled infrared heating system used several electrode-integrated optics/sensors and a custom-made control software to monitor, control and store the surface temperature data in real time. This system used a Class 4 fiber-optic laser (Lasermate IMLN-980–10WFME) with an output beam at 980 nm, which was known to penetrate into tissue at prolonged exposure times with minimal surface damage^21^, to heat the surface of the target. The infrared laser light was sent though an optical fiber imbedded in a 4-needle Teflon electrode, as shown in Fig. 5. The electrode was fixed vertically to the target, allowing the laser beam to be perpendicularly incident onto the target surface. A ZTP-135SR thermopile temperature sensor, mounted inside the Teflon casing of the electrode, reads the surface irradiation in real time by converting the optical signal to an electrical signal that is then translated to a temperature reading using an Arduino Uno-based microcontroller. The temperature data was displayed and recorded by a Graphical User Interface (GUI) created using LabVIEW 2014. To achieve controlled heating, the system reads and stores the temperature trend in real time, compares the current measurement value to a preset target temperature, and modulates the laser heating by turning the laser power on and off accordingly. This system can be operated with electric field strengths up to 35 kV/cm causing minimum electromagnetic interference on the instruments.

The thermopile-based temperature sensor was mounted within the Teflon electrode housing at a viewing angle of 63°, as shown in Fig. 4(B). The 63° viewing angle was necessary to allow the temperature sensor to collect sufficient infrared photons from the surface under normal incidence of the laser beam. To obtain the surface temperature, the sensor was attached to a low-pass filter and amplifying circuit, which filtered out electromagnetic interference produced by high voltage nanosecond pulses and amplified the temperature signal in the form of a low-frequency voltage signal. A similar temperature sensor was used in our previous work^30^.

### System calibration

The voltage supplied by the temperature monitoring circuit was fed into an analog pin on an Arduino Uno microprocessor, where it was converted from a voltage to a temperature reading using a built-in 10-bit Analog to Digital Converter (ADC), which assigns analog voltage values to a discrete digital reading. The trend of these readings can be mapped to a known temperature trend, creating an accurate temperature probe. The temperature calibration was acquired by comparing the readings of the thermopile sensor with a thermocouple (Omega: 5SC-GG-K-30–36) on a hot plate over a range of temperature from 22 °C to 50 °C. During calibration, it is important to keep the thermopile at a constant distance from the calibration target and a constant field of view angle. To ensure accurate calibration, six measurements were performed at 1 °C increment from 22 °C to 50 °C with a field of view angle of 63°. A linear fitting was applied to the averaged data to obtain the following equation that relates the thermopile voltage output to the actual surface temperature:

1 $$T=43.923(V)-22.977$$

where *T* is the calculated surface temperature (°C) and *V* is the measured voltage (mV).

To compensate for the digital instability caused by electronic noise and electromagnetic interference, a data processing algorithm called oversampling and averaging was implemented. Oversampling and averaging works by acquiring a higher number of samples and averaging them together to create a result with higher accuracy^31^. This process allows ADC devices with limited sampling-rate capacity to detect smaller variations in the signal by increasing the number of discrete digital values in each data-sampling so that the averaged value represents the signal variation more accurately. This is particularly useful to the temperature measurements with relatively small temperature changes over a relatively long time, e.g. 0.1 °C temperature increase over one second. It is hence important to resolve the changes by obtaining a higher resolution signal. For every ‘n’ bits of additional resolution needed, a total of 4^n^ samples need to be taken at the hardware-allowed resolution^31,32^. However, since every bit of resolution acquired from this technique requires the acquisition of exponentially more samples, the response time of the system using this new algorithm will decrease drastically as the resolution increases. To calculate the new sampling rate for oversampling, the following relationship is used^31^:

2 $${f}_{os}={4}^{n}\ast {f}_{s}$$

where *n* is the number of additional bits for a resolution desired, ${f}_{s}$ is the max sampling rate of the ADC device and ${f}_{os}$ is the oversampling sampling rate needed to take the higher accuracy data. Note that the sampling rate here is defined as total number of samples per averaged signal (measurement) and ${f}_{s}$ and ${f}_{os}$ are in the unit of samples/data.

In this work, the resolution of the device was increased from 10-bits to 15-bits to provide a temperature measurement with the precision of 0.01 °C. This was realized by averaging 1024 samples at the 10-bit resolution to generate one 15-bit sample. An accuracy of ±1.8 °C temperature measurement was achieved using the device with a system data acquisition rate of 4 samples per second.

### Beam profile determination using knife-edge technique

To determine the effective area treated by the laser, the irradiation distribution, or the beam profile, needed to be determined. A knife-edge technique was employed for beam profiling where a beveled thin blade was placed perpendicular to the laser beam and in front of a power meter, as shown in Fig. 5A^33–35^. With the laser beam incident on the power meter, the edge of the blade was moved transversally across the beam in 50-µm increments and a recording of the irradiation power passing through the blade was taken at each point. The acquired plot shows the two-dimensional Gaussian profile integrated over the displacement of the razor blade, which can be estimated by the following^35^:

3 $${P}_{T}(x)={P}_{T0}r\ast \sqrt{\frac{\pi }{8}}{\rm{erf}}\,\left[\frac{\sqrt{2}x}{r}\right]$$

where ${P}_{T}$ is the total power supplied by the laser (mW), ${P}_{T0}$ is the power read by the power meter (mW), *r* is the beam width (mm), corresponding to the $1/{e}^{2}$ radius of the Gaussian Beam, and *x* is the displacement position of the blade (mm).

The $1/{e}^{2}$ radius of the beam can be estimated by taking a smoothing derivative of the data and fitting it with a Gaussian profile^35^. A 2^nd^ order Gaussian fit was applied to the derivative data, which gave a clean representation of the beam profile, as shown in Fig. 5B,C. Assuming that the beam was uniform in shape, a normalized 2-dimmensional beam profile was created, shown in Fig. 5D.

For synergetic effect tests, it would be ideal that the irradiation covers the entire region between the electrode needles and the highest irradiation intensity is at the center of the needle array, where the electric field is the weakest. The electrode designed for this study has a 5-mm × 7-mm needle spacing, so an irradiation radius ≥2.5 mm would be able to provide sufficient coverage. With that goal in mind, various distances between the fiber and the surface of the target were tested to achieve the optimal irradiation coverage. A gap distance of 2 cm between the laser optic fiber and sample surface results in an irradiation radius, or 1/*e*^2^ radius of 2.7 mm, corresponding to a beam width of 5.4 mm. Since this 2-cm gap distance is longer than the length of the non-inserted portion of the needles, the laser fiber was integrated in the center of the Teflon housing and recessed 8 mm from the Teflon surface. An additional beam profile measurement was performed using the integrated laser fiber and confirmed that the beam shape was not affected by this recessed integration.

### Thermal property measurements of pig skin

To quantify the effects that the heating treatment would have on tissue, several experiments were performed with harvested pig skin, due to its similarities to human skin^36–38^. Specifically, the penetration depth of the laser and the amount of heat that transmitted through the tissue over time were measured through observing the change in the thermal properties of the sample, specifically the absorption coefficient. The absorption coefficient can be approximated using Beer-Lambert’s law^39,40^:

4 $${\boldsymbol{A}}=\frac{{I}_{S}}{{I}_{0}}={e}^{-{\mu }_{a}d}$$

where: ***A*** is the transmittance, ${I}_{S}$ is the transmitted light intensity (mW), ${I}_{0}$ is the initial light intensity (mW), ${\mu }_{a}$ is the absorption coefficient (1/*mm*^−1^) and *d* is the thickness of the skin sample (mm).

The Beer-Lambert’s law approximation is valid under ideal conditions, i.e. the light being perfectly collimated and monochromatic with a uniformly absorbing media^37^. Since biological tissue consists of many components that absorb light nonuniformly, such as multiple tissue layers, tissue types and blood flow, the Beer-Lambert’s law can only provide an approximate and equivalent absorption coefficient of biological samples of relatively similar structures. In this study, the equivalent absorption coefficient for the biological sample is determined by identifying the average absorption coefficient of pig skins of various thicknesses. Transmittance of the skin samples with various thicknesses were measured. The slope of a linear fit of the natural log of the transmittance, ln(***A***), with respect to the sample thickness, *d*, yields the equivalent absorption coefficient ${\mu }_{a}$, according to Eq. (4) or $\mathrm{ln}({\boldsymbol{A}})=-\,{\mu }_{a}d$. Additionally, the penetration depth of the laser light into the sample can be determined using the following relationship:

5 $${\delta }_{p}=\frac{1}{{\mu }_{a}}$$

where: ${\delta }_{p}$ is the penetration depth of light into the sample (mm). This value is vital in determining how much tissue is directly heated by the infrared light and, depending on our sample thickness, how much would need to be heated through thermal diffusion.

The flat face of the sample was held perpendicular to the incident beam and in front of either a power meter, which measures the amount of transmitted light through the sample, or a thermal camera, which measures the spatiotemporal development of the heat transmission through the back of the sample. The laser fiber was positioned in front of the sample at 2 cm and was set to a constant laser power of 2.3 watts for 3 min. When using the power meter to collect data, the sample was placed directly in front of the meter and transmitted light intensity after the tissue sample was recorded every 5 seconds. When using the thermal camera to collect spatiotemporal data, the camera was placed 25 cm away from the sample and a thermal image of the heat transmission at the rear of the sample was acquired every 10 seconds. Both tests were repeated for 5 samples, each with thicknesses ranging from 3.9 mm to 6.8 mm, representing the typical tissue/tumor thickness for the animal studies.

### Treatment protocol for KLN205 tumors in mice

Female DBA/2 mice (6–8 weeks of age), purchased from Jackson Laboratory, were used for the *in vivo* study. For tumor induction, mice were injected with 1 × 10^6^ KLN205 cells in 50 μL Dulbecco’s phosphate buffered saline (DPBS) on the pre-shaven left flank. The size of primary tumor was measured twice a week with digital calipers. Tumor volume was determined using the following formula^17,28^:

6 $$V=\frac{\pi a{b}^{2}}{6}$$

where (a) is the longest diameter (mm) and (b) is the shortest diameter perpendicular to (a) (mm). Mice were euthanized at the end of the follow-up period or when they met criterion described at experimental endpoints, such as large tumor volumes or declining health. All experimental protocols were approved by Old Dominion University Institutional Biosafety Committee (IBC) and Institutional Animal Care and Use Committee (IACUC). All experiments were performed in accordance with relevant guidelines and regulations.

Tumors ranging from 40–100 mm^3^ in size, determined by Eq. (6), were treated with laser heating only, nsPEF only or the combination of nsPEF and heating. Tumor bearing animals without treatment were the control group. Moderate surface heating of tumor with a target temperate 43 °C was achieved by the controlled infrared heating system (Fig. 4). The parameters of nsPEF were a pulse width of 100 ns, 5 pulses per second, 600 pulses and electric field strength of 14 to 30 kV/cm. For the combination treatment, the tumor was preheated to the target temperature 43 °C followed by nsPEF treatment, during which the temperature of tumor was maintained at an average of 43 °C. Once the treatment was complete, the mice were monitored daily and the tumors were measured every 3–4 days.

A preheating protocol was employed here before the nsPEF treatment in order to properly elevate the entire tumor’s temperature to the desired level, not just the surface. Pre-heating would also cause the absorption coefficients to be in the decaying regime, as shown in Fig. 1C, before the nsPEF treatment began, allowing for a more complete elevation of the body temperature. Using an anesthetized mouse for calibration, a thermocouple as inserted underneath the tumor and the heating time needed for the thermocouple to reach 43 °C was measured. A pre-heat time of 1 minute after the surface temperature of 43 °C was reached proved to be sufficient for an even heating of the tumor. Note that the temperature of 43 °C was chosen for this *in vivo* study. After all, only a narrow window of temperature range, i.e. 37–45 °C, could be considered for moderate heating studies. The lowest temperature in this window corresponds to the body temperature (~37 °C) and the highest is the threshold of skin pain sensation (45 °C). As a feasibility test, the modulate heating (43 °C for <2 min) protocol was adopted based on previous studies where such moderate heating significantly enhanced the tumor killing by electric pulses in both *in vitro* and *in vivo* pancreatic cancer models^17^. Nevertheless, studies of different surface temperatures as well as the use of different heating time before and after the treatment would help understand the sensitization of the cells to nsPEFs at different heating conditions. These systematic studies will be incorporated into our future work.
